# Impact of anesthesia methods on adverse cardiovascular events during painless gastroscopy in frail older patients: study protocol for a prospective controlled trial

**DOI:** 10.3389/fmed.2026.1784922

**Published:** 2026-03-05

**Authors:** Xuming Liu, Haijun Hou

**Affiliations:** 1 Department of Anesthesiology, Beijing Friendship Hospital, Capital Medical University, Beijing, China; 2 Department of Molecular Pharmacology, Faculty of Science and Engineering, Groningen Research Institute of Pharmacy, University of Groningen, Groningen, Netherlands; 3 Groningen Research Institute for Asthma and COPD (GRIAC), University Medical Center Groningen, University of Groningen, Groningen, Netherlands; 4 Department of Pain Medicine, Guang’anmen Hospital, China Academy of Chinese Medical Sciences, Beijing, China

**Keywords:** adverse cardiovascular events, anesthetic techniques, frail elderly, frailty, gastroscopy

## Abstract

**Background:**

Frailty is common in older adults and is associated with increased perioperative cardiovascular risk. A growing number of frail elderly patients undergo painless gastroscopy, yet the optimal anesthetic strategy for this high-risk group remains unclear. Both sedation and general anesthesia may attenuate procedural stress but can themselves provoke hemodynamic instability. Robust prospective data comparing the cardiovascular safety of these two approaches in frail older patients are lacking.

**Method:**

This is a single-center, prospective, randomized, controlled, single-blind trial. A total of 226 frail elderly patients (age ≥65 years, FRAIL score ≥3, ASA II-III) scheduled for elective painless gastroscopy will be randomly assigned (1:1) to midazolam-based or propofol-based procedural sedation anesthesia care. Standardized anesthetic protocols and monitoring will be used in both groups. The primary outcome is the incidence of peri-procedural cardiovascular adverse events from the start of endoscopy to discharge from the post-anesthesia care unit (PACU). Secondary outcomes include the incidence of non-cardiovascular adverse events, the incidence of delayed adverse events, postoperative pain intensity, satisfaction levels, the number of intra-procedural supplemental drug doses, the time to recovery of consciousness, and the time to recovery of airway and motor function.

**Discussion:**

This trial aims to provide prospective evidence on the comparative cardiovascular safety of midazolam-based versus propofol-based procedural sedation anesthesia in frail elderly patients undergoing gastroscopy and may help guide individualized, risk-based anesthetic decision-making in this vulnerable population.

**Clinical trial registration:**

ClinicalTrials.gov, identifier NCT06192082

## 1 Introduction

As the global population ages, the number of elderly patients requiring anesthesia for diagnostic and surgical procedures continues to rise. Frailty—a multifactorial geriatric syndrome characterized by reduced physiological reserve and increased vulnerability to stressors—has emerged as a major determinant of perioperative risk. Its prevalence rises steeply with advancing age and is closely associated with accelerated biological aging and complex genetic pathways (1, 2). Accumulating evidence demonstrates that frailty independently predicts adverse perioperative outcomes, including prolonged hospitalization, postoperative delirium, and increased mortality, showing a clear dose-response relationship between frailty severity and poor prognosis (3–5). Accordingly, optimizing anesthetic management for frail elderly patients has become a key priority in contemporary anesthesiology.

Gastrointestinal malignancies are increasingly prevalent among the elderly, and epidemiological evidence suggests that individuals aged 60 years and above constitute over 60% of all gastrointestinal cancer cases worldwide (6, 7). Survival rates for gastric and colorectal cancers decline markedly with age, emphasizing the importance of early diagnosis (8, 9). Endoscopic evaluation remains the gold standard for detecting gastrointestinal tumors. However, the procedure itself can provoke a pronounced sympathetic stress response due to pharyngeal and visceral stimulation, resulting in transient hypertension, tachycardia, or myocardial ischemia (10–12). Frail elderly patients, with their inherently diminished cardiovascular reserve, are particularly susceptible to stress-induced adverse cardiovascular events (ACVEs) such as arrhythmias and ischemic injury. Nationwide data indicate that peri-procedural myocardial infarction and cardiogenic shock carry exceptionally high mortality in this population (13).

Sedation and anesthesia are routinely used to mitigate these hemodynamic responses and improve procedural tolerance. However, anesthetic agents themselves may compromise cardiovascular stability. Commonly used drugs—such as propofol, opioids, and benzodiazepines—can induce dose-dependent hypotension, bradycardia, and respiratory depression (13). Consequently, anesthesiologists face a delicate balance: sedation may inadequately suppress the stress response, while general anesthesia increases the risk of hemodynamic instability. Despite ongoing debate, evidence remains insufficient to define the optimal anesthetic strategy for frail elderly patients undergoing painless gastroscopy.

In light of the above, we designed a prospective randomized controlled trial to compare a midazolam-based regimen (sufentanil plus midazolam) with a propofol-based regimen (sufentanil plus propofol) in frail older adults undergoing painless gastroscopy. We hypothesize that anesthesia type significantly affects the incidence of peri-procedural ACVEs and overall procedural safety.

## 2 Methods

### 2.1 Study design

This is a prospective, randomized, controlled, single-blind clinical trial conducted by the Department of Anesthesiology, Beijing Friendship Hospital, Capital Medical University. The study has been approved by the institutional Bioethics Committee (approval number: 2023-P2-301-01) and registered at ClinicalTrials.gov (identifier: NCT06192082).

A total of 226 patients aged 65 years or older scheduled for painless gastroscopy will be enrolled. The study aims to evaluate the effects of different anesthesia methods—midazolam-based vs. propofol-based regimen—on peri-procedural cardiovascular safety in frail elderly patients. All study procedures will be conducted in accordance with the Declaration of Helsinki and relevant Good Clinical Practice (GCP) guidelines.

### 2.2 Randomization and blinding

**Figure 1:**
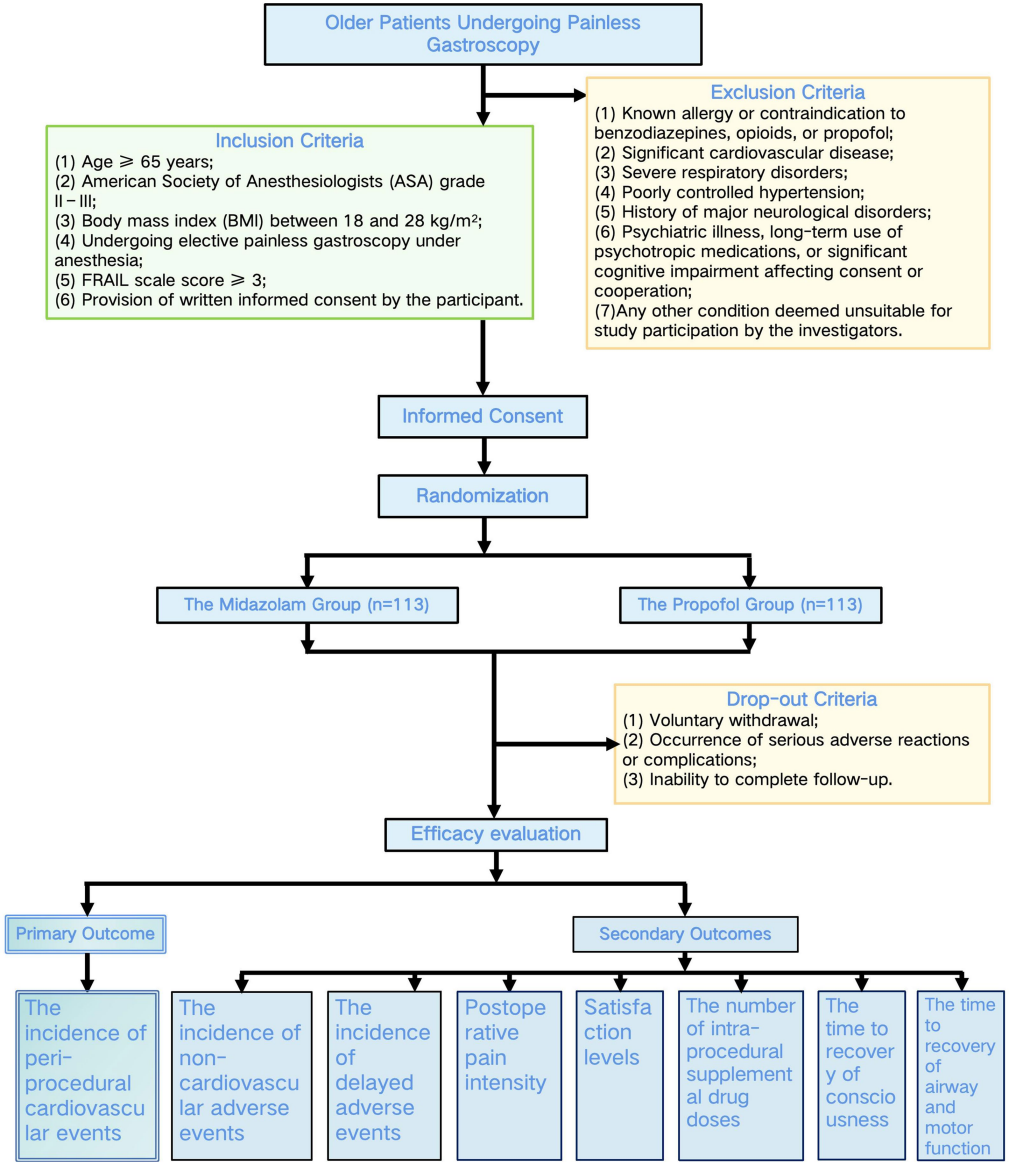
Study flowchart.

Participants will be randomly assigned in a 1:1 ratio to either the midazolam group (Group A) or the propofol group (Group B) using a computer-generated random number sequence (Figure 1). The randomization list will be generated by an independent statistician who is not involved in patient recruitment or anesthesia management. Group allocation numbers will be printed and placed in sequentially numbered, opaque, sealed envelopes.

When an eligible patient arrives at the endoscopy center, an anesthesiologist will conduct a preoperative interview, explain the study details, and obtain written informed consent. The anesthesiologist will then open the next envelope in sequence and assign the patient to the designated group, and record the baseline data before anesthesia induction.

This study will adopt a single-blind design: the anesthesiologist administering anesthesia will be aware of group allocation, whereas the endoscopist, patients, and outcome assessors (including postoperative follow-up investigators) will remain blinded to group assignment. In the event of a medical emergency or serious adverse event, the blinding may be broken to ensure patient safety, and the reason for unblinding will be documented in the case report form (CRF).

To enhance patient engagement and compliance, small appreciation gifts (e.g., a keychain, fruit, or a healthy beverage; approximate value: 20 RMB/~3 USD) will be provided to participants after completion of the study procedures.

### 2.3 Study population

A total of 226 patients scheduled for elective painless gastroscopy will be recruited according to the following criteria.

#### 2.3.1 Inclusion criteria

(1) Age ≥65 years.
(2) American Society of Anesthesiologists (ASA) grade II-III.
(3) Body mass index (BMI) between 18 and 28 kg/m^2^.
(4) Undergoing elective painless gastroscopy under anesthesia.
(5) FRAIL scale (14) score ≥3.
(6) Provision of written informed consent by the participant.

#### 2.3.2 Exclusion criteria

(1) Known allergy or contraindication to benzodiazepines, opioids, or propofol.
(2) Significant cardiovascular disease, including acute heart failure, unstable angina, myocardial infarction within the past 6 months, heart rate (HR) <50 bpm, third-degree atrioventricular block, severe arrhythmia, or moderate-to-severe valvular heart disease.
(3) Severe respiratory disorders, such as acute respiratory infection, acute exacerbation of chronic obstructive pulmonary disease, or uncontrolled asthma.
(4) Poorly controlled hypertension.
(5) History of major neurological disorders, including craniocerebral injury, intracranial hypertension, cerebral aneurysm, cerebrovascular accident, or other central nervous system diseases.
(6) Psychiatric illness, long-term use of psychotropic medications, or significant cognitive impairment affecting consent or cooperation.
(7) Any other condition deemed unsuitable for study participation by the investigators.

#### 2.3.3 Drop-out criteria

Participants may be withdrawn from the study under any of the following circumstances:

(1) Voluntary withdrawal.
(2) Occurrence of serious adverse reactions or complications.
(3) Inability to complete follow-up.

### 2.4 Procedure and intervention

Before enrollment, all potential participants will be screened according to the predefined inclusion and exclusion criteria. Eligible patients will then be approached during the preoperative visit, where written informed consent will be obtained, followed by documentation of baseline demographic data and the FRAIL scale score (Table 1). A detailed medical history will be taken to exclude contraindications such as soybean allergy and a family history of malignant hyperthermia. All patients will be required to fast for at least 12 h and abstain from liquids for 8 h prior to the procedure.

**Table 1:** FRAIL scale.

Item	Question	Yes (1 point)	No (0 points)
1	During the past 4 weeks, have you felt tired most of the time or every day?	□	□
2	Do you have difficulty climbing one flight of stairs without resting or using assistive devices?	□	□
3	Do you have difficulty walking one block (≈100 m) without assistive devices?	□	□
4	Do you have five or more chronic medical conditions?	□	□
5	Have you unintentionally lost more than 5% of your body weight in the past year?	□	□
Total score			

The FRAIL scale consists of five yes/no items (0–5 points); a total score ≥3 indicates frailty.

Upon entering the endoscopy suite, an upper-limb intravenous line will be established, and lactated Ringer’s solution will be infused at a rate of 3 mL/kg/h. Standard monitoring will be initiated, including electrocardiography (ECG), non-invasive blood pressure (NIBP), HR, and peripheral oxygen saturation (SpO_2_). An appropriately sized face mask will be selected according to the patient’s facial contour, and the mask cushion will be adjusted based on cheek fullness to ensure an airtight seal when secured with a head strap.

The adjustable pressure-limiting valve of the anesthesia machine will be set to fully open, and 100% oxygen will be delivered at a fresh gas flow of 8 L/min. Patients will be instructed to breathe calmly. Effective pre-oxygenation will be considered achieved when the end-tidal oxygen concentration reaches 88%–90%.

#### 2.4.1 The midazolam group (Group A)

Participants allocated to Group A will receive intravenous sedation. Five minutes prior to endoscope insertion, sufentanil will be administered intravenously at a dose of 0.05–0.1 μg/kg over 30 s. Two minutes before the start of the procedure, midazolam will be administered intravenously at a dose of 0.02 mg/kg. Sedation depth will be evaluated using the Modified Observer’s Assessment of Alertness/Sedation (MOAA/S) scale (15). Endoscope insertion will be initiated when the MOAA/S score reaches 2-3.

If the patient exhibits coughing, agitation, body movement, or other signs of intolerance during the procedure, an additional bolus of midazolam 0.02 mg/kg may be administered, followed by reassessment of the MOAA/S score. The procedure will continue once adequate sedation (MOAA/S 2-3) is re-established.

#### 2.4.2 The propofol group (Group B)

Participants assigned to Group B will receive intravenous general anesthesia. Five minutes prior to endoscope insertion, sufentanil will be administered intravenously at a dose of 0.05–0.1 μg/kg over 30 s. Two minutes before the start of the procedure, propofol will be administered at an induction dose of 1 mg/kg. Anesthesia will then be maintained using a continuous infusion of propofol at 3 mg/kg/h. Endoscope insertion will be performed once the MOAA/S score reaches 0-1.

If the patient exhibits intolerance during the procedure, an additional bolus of propofol 0.5 mg/kg may be administered, followed by reassessment of the MOAA/S score. The procedure will continue once adequate anesthesia (MOAA/S 0-1) is re-established.

All anesthetic agents will be discontinued before the end of the procedure. From the start of endoscopy, NIBP will be measured at 2 min intervals, and HR, SpO_2_, and ECG will be continuously monitored until the endoscope is withdrawn from the oral cavity.

After the procedure, patients will be transferred to the PACU for continued monitoring until they achieve adequate recovery, defined as a Steward score of > 4. Before discharge from the PACU, satisfaction ratings will be obtained from the endoscopist, the attending anesthesiologist, and the patient.

All intraoperative and PACU adverse events will be documented in real time and managed in accordance with current clinical guidelines. In the event of hypoxemia (SpO_2_ < 90%), immediate assisted ventilation with positive-pressure oxygen will be initiated. An experienced anesthesiologist will be responsible for the anesthesia management of each patient and may modify the anesthetic plan based on the patient’s clinical status and safety considerations. Any protocol deviations or dose adjustments will be recorded in detail.

### 2.5 Study outcomes

#### 2.5.1 The primary outcome

The primary outcome is the incidence of peri-procedural cardiovascular events occurring from the start of the endoscopic examination until transfer to PACU.

Cardiovascular events are defined as any of the following: HR deviation exceeding ±30% from baseline; blood pressure fluctuation exceeding ±30% from baseline; clinical manifestations of myocardial ischemia, such as angina pectoris or myocardial infarction; cardiac arrest; acute heart failure; new-onset or worsening arrhythmia; myocardial ischemia confirmed by ECG or clinical assessment; or pulmonary embolism.

#### 2.5.2 The secondary outcome

Secondary outcomes include the incidence of non-cardiovascular adverse events, the incidence of delayed adverse events, postoperative pain intensity, satisfaction levels, the number of intra-procedural supplemental drug doses, the time to recovery of consciousness, and the time to recovery of airway and motor function.

(1) Non-cardiovascular adverse events will be monitored from the beginning of the procedure until discharge from the PACU and will include hypoxemia (SpO_2_ < 90%), respiratory apnea (defined as cessation of airflow for ≥10 s), body movement, procedural intolerance, coughing, aspiration, muscle tremor, nausea and vomiting, or dyspnea.
(2) Delayed adverse events will be assessed through a structured telephone follow-up on postoperative day 3, including the occurrence of dizziness, headache, palpitations, dyspnea, reduced mobility, and nausea or vomiting.
(3) Postoperative pain will be assessed in the PACU once the patient has fully regained consciousness. Pain intensity will be evaluated using the Visual Analog Scale (VAS), where 0 indicates no pain and 10 represents the worst pain imaginable.
(4) Satisfaction will be assessed using a Verbal Rating Scale (VRS) from 1 to 10, where 1 indicates very dissatisfied and 10 indicates very satisfied. For the purposes of statistical analysis, scores will be categorized into three levels: 1–4 = poor satisfaction; 5–7 = moderate satisfaction; 8–10 = good satisfaction. This categorization is based on prior usage of 0–10 scales in pain and satisfaction research (16), adapted for this study.
(5) The time to recovery of consciousness will be defined as the interval from the end of the procedure to the moment when the MOAA/S score first returns to 5. Consciousness will be assessed every 2 min using the MOAA/S scale: 5 = prompt response to normal verbal command; 4 = delayed response to normal verbal command; 3 = response only after loud or repeated calling; 2 = response only to mild physical stimulation (e.g., shoulder shaking); 1 = response only to trapezius muscle squeeze; 0 = no response to trapezius muscle squeeze. The time point at which a score of 5 is reached will be recorded as the recovery time.
(6) The time to recovery of airway and motor function will be defined as the interval required for the Steward recovery score to reach >4 (17). The detailed scoring criteria are shown in Table 2.

**Table 2:** Steward recovery score.

Item	Description	Score
Level of consciousness	Fully awake	2
	Responds to verbal stimuli	1
	No response to stimulation	0
Airway patency	Coughs effectively	2
	Maintains airway independently	1
	Requires airway support	0
Motor activity	Spontaneous purposeful movement	2
	Non-purposeful movement	1
	No movement	0

### 2.6 Management of intra-procedural adverse events and safety procedures

Adverse events occurring during the endoscopic procedure will be monitored continuously and managed according to predefined criteria. The following events and corresponding interventions will be recorded:

(1) Respiratory depression, defined as a respiratory rate <8 breaths/min, will be managed with continuous oxygen supplementation and close monitoring to maintain SpO_2_ ≥ 90%.
(2) Apnea, defined as cessation of breathing for ≥10 s, will be managed with assisted ventilation and airway support as needed.
(3) Hypoxemia, defined as SpO_2_ 75–89% for <60 s (mild), SpO_2_ 75–89% for >60 s (moderate), or SpO_2_ < 75% (severe). Management includes oxygen supplementation, jaw thrust, positional adjustment, and placement of a nasopharyngeal airway if required to relieve tongue obstruction.
(4) Hypertension, defined as an increase in blood pressure >20% above baseline, may be treated with intravenous urapidil 0.1 mg/kg at the discretion of the attending anesthesiologist.
(5) Hypotension, defined as a decrease in blood pressure >20% from baseline, may be treated with intravenous ephedrine 0.1 mg/kg as clinically indicated.
(6) Tachycardia, defined as HR >100 bpm, may be managed with intravenous esmolol 0.15–0.3 mg/kg.
(7) Bradycardia, defined as HR <50 bpm, may be treated with intravenous atropine 0.01 mg/kg.

Any adverse events spontaneously reported by the patient or observed by investigators will be documented. A serious adverse event is defined as any untoward medical occurrence that results in death, is life-threatening, requires hospitalization or prolongation of existing hospitalization, results in persistent disability, causes a congenital anomaly, or requires urgent intervention to prevent such outcomes. The causal relationship between the event and the study intervention will be assessed by the investigators.

All serious adverse events will be recorded in detail and reported to the institutional ethics committee within 24 h.

### 2.7 Data collection and monitoring

Baseline demographic data and the FRAIL score will be collected during the pre-anesthesia evaluation by the attending anesthesiologist. Intra-procedural cardiovascular and non-cardiovascular adverse events, pain assessment, satisfaction ratings, the number of supplemental sedative doses, intra- and postoperative sedation scores, and the time to recovery of airway and motor function will be documented in real time by the anesthesiologist responsible for the case. Data obtained in the PACU, including recovery parameters, pain intensity, and satisfaction scores, will also be recorded by the same anesthesiologist. Postoperative follow-up on day 3 will be conducted via telephone by study team members who are blinded to group allocation.

All study data will be documented in the CRF. Missing or incomplete data will be recorded together with the reason. Baseline characteristics, intra-procedural variables, PACU observations, and postoperative follow-up outcomes will be collected according to the predefined schedule.

A Data Monitoring Committee (DMC), independent of the investigators and sponsor, will oversee data quality, review adverse events, and supervise protocol compliance. The DMC will conduct audits every 6 months and determine whether protocol amendments are necessary. Any revisions will be submitted to the ethics committee and updated on the clinical trial registry.

The chief anesthesiologist will perform monthly internal reviews to ensure data accuracy and adherence to the study protocol. No interim analysis is planned, and unblinding will occur only if required for patient safety.

All original study documents, including informed consent forms and CRFs, will be securely stored in locked cabinets and password-protected databases for at least 5 years, after which they will be destroyed according to institutional policy. De-identified data will not be publicly accessible until the study results are published.

### 2.8 Sample size calculation

The sample size was calculated using PASS 11.0. Preliminary data suggested an incidence of peri-procedural cardiovascular adverse events of 37% in the Midazolam group and 20% in the Propofol group. With a two-sided significance level (*α*) of 0.05, a statistical power of 80%, and an allocation ratio of 1:1, a minimum of 102 participants per group was required to detect a clinically meaningful difference. To account for an anticipated dropout rate of 10%, the final target sample size was set at 226 participants (113 per group).

### 2.9 Statistical analysis

Statistical analyses will be performed using SPSS 24.0. All efficacy analyses will follow the intention-to-treat (ITT) principle, including all randomized participants. Safety analyses will be conducted in the safety population, defined as all participants who receive any study-related medication. A two-sided *p*-value <0.05 will be considered statistically significant.

Continuous variables will be tested for normality using the Shapiro-Wilk test. Normally distributed data will be presented as mean ± standard deviation and compared using the independent-samples *t* test or repeated-measures Analysis of Variance (ANOVA) where appropriate. Non-normally distributed data will be summarized as median (interquartile range) and analyzed using the Mann-Whitney *U* test or Friedman test. Categorical variables will be expressed as frequencies (percentages) and analyzed using the Chi-square test or Fisher’s exact test, as appropriate.

To evaluate the independent effect of anesthetic technique on the incidence of peri-procedural cardiovascular events, multivariate logistic regression will be performed with adjustment for pre-specified covariates (e.g., age, sex, BMI, ASA status, comorbidities). Results will be reported as odds ratios with 95% confidence intervals. Pre-specified subgroup analyses of the primary endpoint may be conducted stratified by age group, procedure duration, frailty severity, or other clinically relevant factors.

Missing data for the primary endpoint will be handled using complete-case analysis; no imputation is planned. Sensitivity analysis will be performed by comparing the ITT and per-protocol populations. No interim analysis will be conducted, and no alpha-spending adjustment is required.

## 3 Discussion

Frailty is a common geriatric syndrome with high prognostic value and has been widely recognized as a key determinant of perioperative risk in older adults (18, 19). With the rising incidence of gastrointestinal malignancies and other digestive diseases, gastrointestinal endoscopy is increasingly performed as a crucial tool for screening and treatment in the elderly, leading to a growing number of frail patients undergoing endoscopic procedures under sedation or anesthesia. Because these patients have limited physiological reserve and multiple comorbidities, perioperative management-particularly the choice of anesthetic technique-is especially challenging and requires a careful balance between procedural conditions, hemodynamic stability, and overall safety. Accumulating evidence indicates that frailty is not only associated with poor perioperative outcomes in general (20), but is also closely linked to cardiovascular instability and major cardiovascular events (21, 22). Therefore, developing safer and more individualized anesthesia strategies for frail elderly patients undergoing gastrointestinal endoscopy is of considerable clinical importance and urgency.

Although both sedation and propofol-based general anesthesia are widely used for painless gastroscopy, neither technique is without risk. Moderate-to-deep sedation can effectively reduce procedural discomfort and anxiety, shorten recovery time, and decrease resource utilization, and is therefore recommended as a first-line strategy for many routine gastrointestinal endoscopic procedures (23). However, sedative agents-particularly opioids-exert dose-dependent cardiopulmonary depressant effects and may lead to hypotension, bradycardia, respiratory depression, apnea, and hypoxemia, which occur more frequently in elderly and frail patients (11, 24). In addition, deep sedation may unpredictably drift toward general anesthesia, requiring timely airway interventions by anesthesia providers with advanced airway management skills.

In contrast, general anesthesia offers more complete immobility, improved operating conditions, and potentially better airway protection, which may be advantageous in complex therapeutic endoscopy or in selected high-risk patients (24). Nevertheless, it is typically associated with deeper cardiovascular and respiratory suppression, the need for airway devices, more intensive monitoring, and greater resource consumption (25). Although numerous retrospective and registry studies have examined different sedative strategies, most have included mixed populations with substantial variation in age and frailty, and only limited prospective data are available in frail elderly patients undergoing painless gastroscopy. Our randomized trial aims to complement the existing literature by exploring the cardiovascular safety profiles of sedation and general anesthesia in this specific population.

In this context, this trial adopts a prospective, randomized, single-blind design to compare two standardized anesthetic regimens in frail older adults undergoing painless gastroscopy. Restricting inclusion to patients with a FRAIL score ≥3 helps to delineate a clearly defined high-risk cohort and may increase the clinical applicability of the findings. The sample size has been calculated on the basis of preliminary cardiovascular event rates to provide adequate statistical power, and stratified randomization is expected to limit baseline imbalances between groups. In addition, the use of structured intra- and post-operative monitoring, together with patient-centered outcomes such as satisfaction and recovery quality, is intended to support both methodological robustness and clinical relevance.

Nonetheless, certain limitations warrant consideration. The single-center design may restrict generalizability, and subjective measures such as satisfaction scoring may introduce response bias. In addition, although powered to detect differences in cardiovascular events, the study may not detect rare but severe complications. The focus on short-term outcomes also precludes evaluation of longer-term effects, including cognitive decline, which is of particular importance in frail elderly populations.

In conclusion, this randomized controlled trial is expected to provide high-quality evidence comparing the cardiovascular safety of sedation and general anesthesia in frail elderly patients undergoing painless gastroscopy. The findings may guide anesthesiologists in making individualized, risk-based decisions and contribute to the development of optimized perioperative management strategies for this rapidly growing patient population.

## 4 Trial status

This protocol has been submitted for ethical review, and approval was obtained by 10 November 2023. The first subject was successfully recruited on 22 March 2024. As of February 2026, 102 participants have been enrolled at Beijing Friendship Hospital, Capital Medical University. The current protocol version is V1.2/2023-10-19, and if we need to update it in the future, we will submit amendments to the ethics committee and clinical trial registry.
