# Supplementary material for: Impact of anesthesia methods on adverse cardiovascular events during painless gastroscopy in frail older patients: study protocol for a prospective controlled trial
Source: Front Med (Lausanne). 2026 Mar 5;13:1784922. doi: 10.3389/fmed.2026.1784922 (PMC12999575; doi:10.3389/fmed.2026.1784922)
Supplement: Supplementary file 1 [file Table_1.doc]

Figure . Standard protocol items: study schedule

|  | **STUDY PERIOD** | | | | | | | |
| --- | --- | --- | --- | --- | --- | --- | --- | --- |
|  | **Enrolment** | **Allocation** | | | | **Post-allocation** | | **Close-out** |
| **TIMEPOINT** | **Preoperative**  **visit** | **Before anesthesia** | **Before endoscopy** | **During anesthesia/ endoscopy** | **At the end of endoscopy** | **Day 0**  **(PACU)** | **Day 3** | **Discharge** |
| **ENROLMENT:** | | | | | | | | |
| **Eligibility screen** | X |  |  |  |  |  |  |  |
| **Informed consent** | X |  |  |  |  |  |  |  |
| ***Record basic characteristics*** |  | X |  |  |  |  |  |  |
| **Allocation** |  | X |  |  |  |  |  |  |
| **INTERVENTIONS:** | | | | | | | | |
| ***The Sedation Group*** |  |  |  |  |  |  |  |  |
| ***The Intravenous General Anesthesia Group*** |  |  |  |  |  |  |  |  |
| **ASSESSMENTS:** | | | | | | | | |
| ***MOAA/S score*** |  |  |  |  |  |  |  |  |
| ***The incidence of peri-procedural cardiovascular events*** |  |  |  |  |  |  |  |  |
| ***The incidence of non-cardiovascular adverse events*** |  |  |  |  |  |  |  |  |
| ***The incidence of delayed adverse events*** |  |  |  |  |  |  | X |  |
| ***Postoperative pain intensity*** |  |  |  |  |  | X |  |  |
| ***Satisfaction levels*** |  |  |  |  |  | X |  |  |
| ***The number of intra-procedural supplemental drug doses*** |  |  |  | X |  |  |  |  |
| ***The time to recovery of consciousness*** |  |  |  |  |  | X |  |  |
| ***The time to recovery of airway and motor function*** |  |  |  |  |  | X |  |  |
